# Integrated hybrid sensing and microenergy for compact active microsystems

**DOI:** 10.1038/s41378-022-00393-z

**Published:** 2022-06-06

**Authors:** Hai-Tao Deng, Zhi-Yong Wang, Yi-Lin Wang, Dan-Liang Wen, Xiao-Sheng Zhang

**Affiliations:** School of Electronic Science and Engineering, University of Electronic Science and Technology of China, 611731 Chengdu, China

**Keywords:** Electrical and electronic engineering, Electronic devices

## Abstract

Wearable electronics, as essential components of the Internet of Things (IoT), have attracted widespread attention, and the trend is to configure attractive wearable smart microsystems by integrating sensing, powering, and other functions. Herein, we developed an elastic hybrid triboelectric–electromagnetic microenergy harvester (named EHTE) to realize hybrid sensing and microenergy simultaneously. This EHTE is a highly integrated triboelectric nanogenerator (TENG) and electromagnetic nanogenerator (EMG). Based on the triboelectric–electromagnetic hybrid mechanism, an enhanced electrical output of the EHTE was achieved successfully, which demonstrates the feasibility of the EHTE for microelectronics powering. Moreover, with the merits of the EMG, the developed hybrid microenergy harvester integrated both active frequency sensing and passive inductive sensing capabilities. Specifically, the almost linear correlation of the electromagnetic outputs to the frequencies of the external stimulus endowed the proposed EHTE with an outstanding active frequency sensing ability. In addition, due to the unique structural configuration of the EMG (i.e., a conductive permanent magnet (PM), hybrid deformation layer, and flexible printed circuit board (FPCB) coil), an opportunity was provided for the developed EHTE to serve as a passive inductive sensor based on the eddy current effect (i.e., a form of electromagnetic induction). Therefore, the developed EHTE successfully achieved the integration of hybrid sensing (i.e., active frequency sensing and passive inductive sensing) and microenergy (i.e., the combination of electromagnetic effect and triboelectric effect) within a single device, which demonstrates the potential of this newly developed EHTE for wearable electronic applications, especially in applications of compact active microsystems.

## Introduction

As one of the essential components of next-generation information technologies, the Internet of Things (IoT)^1,2^ is regarded as an important component for the development of modern society in various sectors, including industry, economic activity, transportation, and security. Although the IoT has experienced advanced development in recent years, challenges still exist in meeting the increasing demands on quality of life, especially for the energy supply problems of the IoT sensing network. As the key interface media between the environment and client, the sensing network consists of trillions of distributed sensors for environmental stimulus collection and transduction, in which the power consumption of individual sensors is low but the energy requirements of the total sensing network can be large, on the order of billions of watts. Thus, how to sustainably power sensing networks has become an urgent issue for the further development of the IoT.

One of the most effective approaches is harvesting micro/nano energy from the ambient environment, where diverse energy sources are widely distributed in various forms, such as solar, heat, wind, and vibration. Therefore, a massive effort has been devoted to developing different kinds of micro/nano energy harvesters based on different energy harvesting technologies, including photovoltaic nanogenerators (PVNGs)^3–5^, thermoelectric generators (ThEGs)^6–8^, electromagnetic generators (EMGs)^9–11^, piezoelectric nanogenerators (PENGs)^12–14^, triboelectric nanogenerators (TENGs)^15–19^, and many others^20–23^. However, different kinds of energy harvesting technology have unique working conditions and output properties^24^. For example, light and temperature differences are indispensable working conditions for PVNGs and ThEGs, respectively, but their DC outputs provide a great advantage for wearable electronics powering. For EMGs, the bulk permanent magnet (PM) and coil are two essential components to realize EMGs with high output, but bulky and heavy EMGs are inconvenient to wear. Although flexible EMGs using ferroelastomer materials or flexible coils have already been proposed to address weight and size issues, the output was sacrificed. In addition, organic or inorganic piezoelectric materials and complex manufacturing processes, e.g., polarization treatment, should be required to form PENGs. In comparison, TENGs, with advantages such as no material limitations, high output power and high energy conversion efficiency, have attracted more attention in the IoT, particularly in portable and wearable electronic fields. Thus, great effort has been made to develop different kinds of high-performance TENGs, serving as robust power sources^25–28^ and/or self-powered sensors^29–31^. With the proposal of hybrid energy harvesters, the potential of TENGs has been explored in recent years^32^. By integrating a TENG with other energy harvesting technologies in a single device, e.g., hybrid triboelectric–photovoltaic nanogenerators^33–35^, hybrid triboelectric–piezoelectric nanogenerators^36–38^, hybrid triboelectric–electromagnetic nanogenerators^39–43^, hybrid triboelectric–piezoelectric–pyroelectric nanogenerators^44^, etc.^45^, multiplex electrical outputs can be generated from a single stimulus input, thus enhancing the energy conversion efficiency and enlarging the total output power of these devices. Moreover, hybrid devices can realize functional integration or complementarity. For example, Guo et al. developed all-fiber hybrid piezoelectric-enhanced triboelectric nanogenerators that not only achieved enhanced electrical output but also active motion sensing for real-time fall detection^36^. Wan et al. proposed a flexible hybridized electromagnetic–triboelectric nanogenerator for mechanical energy harvesting and 3D trajectory sensing^42^. Wang et al. demonstrated a complementary electromagnetic–triboelectric active sensor for detecting triggering force and velocity simultaneously.

In this work, we developed an elastic hybrid triboelectric–electromagnetic microenergy harvester (named EHTE), which not only adopted the advantages of several triboelectric–electromagnetic hybrid microenergy harvesters with hybrid and enhanced outputs but also highly integrated active frequency sensing and passive inductive pressure sensing within a single device. Based on the coupling of the triboelectric effect and electromagnetic effect, the EHTE exhibited remarkable triboelectric and electromagnetic outputs and hybrid output for microelectronics powering. This EHTE was successfully demonstrated to directly/indirectly power light-emitting diode (LED) screens and calculators, revealing the attractive potential for micro/nano powering. For hybrid sensing, the proposed EHTE could achieve active frequency sensing in terms of the linear correlation of electromagnetic outputs to frequencies of the external stimulus. In addition, due to the unique structural configuration of the electromagnetic nanogenerator of the EHTE, the feasibility of serving as an inductive sensor for passive sensing is provided. Herein, the inductive response behaviors of the proposed EHTE are caused by the eddy current effect^46,47^, which is a form of electromagnetic induction. Thus, with the merits of the hybridization of this triboelectric nanogenerator and electromagnetic nanogenerator, this newly developed EHTE successfully realized the integration of hybrid sensing (i.e., active frequency sensing and passive inductive sensing) and microenergy (i.e., triboelectric–electromagnetic hybrid microenergy) in compact form, exhibiting promising potential for active microsystem applications.

## Results and discussion

### Characterization of the developed EHTE

**Fig. 1:**
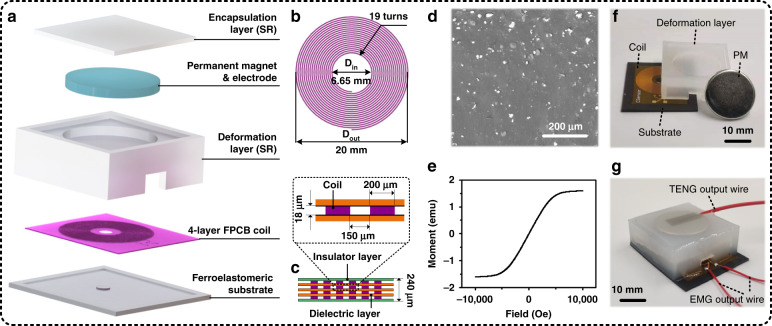
Physical characteristics of the developed elastic hybrid triboelectric–electromagnetic microenergy harvester (EHTE). **a** Explosion view of the hybrid microenergy harvester, consisting of an FeSiAl/SR ferroelastomeric substrate, a 4-layer FPCB coil, an SR-air hybrid deformation layer, a PM and an SR encapsulation layer. **b**, **c** Feature sizes of the 4-layer FPCB coil. **b** Top view of one of the layers of the FPCB coil. Each coil has an outer diameter of 20 mm, an inner diameter of 6.65 mm, and 19 turns. **c** Cross-sectional view of the 4-layer FPCB coil, in which the thickness of the 4-layer FPCB coil and the copper trace are 240 and 18 μm, respectively, the width and space of the trace are 200 and 150 μm, respectively, and the row material of the insulator layer and dielectric layer of the 4-layer FPCB coil is polyimide (PI). **d**, **e** Physical characteristics of the prepared FeSiAl/SR ferroelastomeric substrate with 50 wt% FeSiAl particles. **d** Surface morphology of the FeSiAl/SR ferroelastomer. **e** Magnetic hysteresis loop of the FeSiAl/SR ferroelastomer. Photographs of **f** the discrete components of the hybrid microenergy harvester, i.e., FeSiAl/SR ferroelastomeric substrate embedded with a 4-layer FPCB coil, SR deformation layer and PM, and **g** an assembled hybrid microenergy harvester.

Figure 1a illustrates an exploded view of the elastic hybrid triboelectric–electromagnetic microenergy harvester (EHTE), which consists of an FeSiAl/silicone rubber (FeSiAl/SR) ferroelastomeric substrate, a 4-layer FPCB coil (FPCB is the acronym for flexible printed circuit board), a hybrid silicone rubber–air (SR-air) deformation layer, a permanent magnet (PM) and a silicone rubber (SR) encapsulation layer. The 4-layer FPCB coil, hybrid SR-air deformation layer and PM were the main components of the triboelectric nanogenerator of the EHTE, in which the polyimide (PI) on the top surface of the 4-layer FPCB coil and SR on the bottom surface of the deformation layer were used as triboelectric pairs, and the PM was used as the electrode. Moreover, the FeSiAl/SR ferroelastomeric substrate, 4-layer FPCB coil, hybrid SR-air deformation layer and PM formed the electromagnetic nanogenerator of the EHTE. These discrete components have unique properties. As shown in Fig. 1a, this deformation layer had different grooves on its upper and lower layers. The upper layer was used to fix the PM, and the lower layer was mainly used as an air medium layer for the triboelectric nanogenerator. Notably, the sheet resistance of the PM was 0.36 MΩ/sq, and its magnetic field intensity was ~110.63 mT. As a result, the PM served as the conductive electrode of the triboelectric nanogenerator as well as the magnet of the electromagnetic nanogenerator in the hybrid microenergy harvester. For the 4-layer FPCB coil, Fig. 1b illustrates the top view of one of the layers of the FPCB coil. Each layer had an outer diameter of 20 mm, an inner diameter of 6.65 mm and 19 turns. This means that the total number of turns of the FPCB coil was 76. There was a 3 mm-diameter hole in the 4-layer FPCB coil, which was filled by the FeSiAl/SR ferroelastomer to improve the magnetic flux of the coil. Figure 1c illustrates the cross-sectional view of the 4-layer FPCB coil. The thickness of the whole FPCB coil was 240 μm, the thickness of the copper trace was 18 μm, and the width and space of the copper trace were 200 and 150 μm, respectively. The FeSiAl/SR ferroelastomeric substrate was another crucial component of the hybrid microenergy harvester. Figure 1d shows the surface morphology of the ferroelastomeric substrate with a 50 wt% proportion of FeSiAl particles, in which the FeSiAl particles were well distributed within the silicone rubber matrix. It is noted that the smaller FeSiAl particles observed in the ferroelastomer are due to particles that were semi/fully embedded in the silicone rubber matrix. To confirm the ferromagnetic property of the FeSiAl/SR ferroelastomeric substrate, the magnetic hysteresis loop of the substrate was measured using a vibrating sample magnetometer, as illustrated in Fig. 1e. This result indicated that FeSiAl/SR had inherent properties of ferromagnetic materials, such as high permeability, low remnant magnetization and low coercivity. In addition, a comparison of the characteristics of the FeSiAl/SR ferroelastomers with different proportions of FeSiAl particles was performed, including the surface morphologies of pure FeSiAl particles and FeSiAl/SR ferroelastomers with 25 wt%, 50 wt%, and 75 wt% proportions of FeSiAl particles, as shown in Fig. S1 of the supplementary file, and the magnetic hysteresis loops of the FeSiAl/SR ferroelastomers with 25 wt%, 50 wt%, and 75 wt% proportions of FeSiAl particles, as shown in Fig. S2 of the supplementary file. Figure 1f illustrates the fabricated samples of the main discrete components of the hybrid microenergy harvester, i.e., the FeSiAl/SR ferroelastomeric substrate, 4-layer FPCB coil, hybrid SR-air deformation layer and PM. An assembled sample of the hybrid microenergy harvester is also shown in Fig. 1g.

### Microenergy harvesting based on the triboelectric–electromagnetic mechanism

#### Triboelectric–electromagnetic hybrid mechanism

**Fig. 2:**
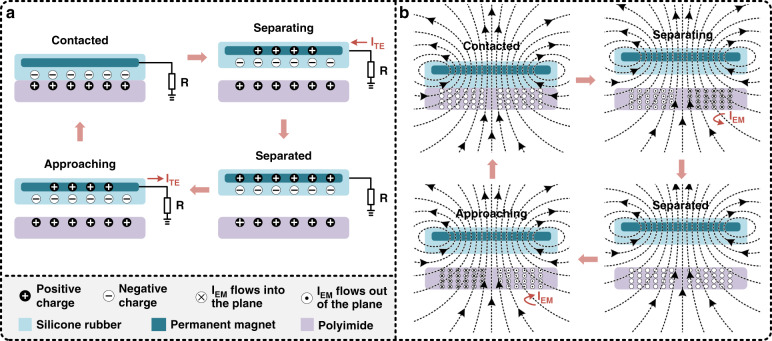
Schematic diagram of the work mechanism of the developed elastic hybrid triboelectric–electromagnetic microenergy harvester (EHTE), which includes two parts. Based on the triboelectric effect and electromagnetic effect, triboelectric and electromagnetic outputs can be simultaneously generated from a single mechanical input. **a** Schematic view of the working mechanism of the TENG, i.e., the coupling of triboelectricity and electrostatic induction, which mainly includes four steps. In the initial state, the device is in the electrostatic neutral state. When the external force makes the lower surface of the deformation layer and the upper surface of the FPCB come into close contact, triboelectric charges are generated at the interface of the SR-PI triboelectric pair due to the triboelectrification effect. When the SR leaves the surface of PI, an internal electric potential can be established to accumulate charges on the PM electrode resulting from electrostatic induction. The accumulated charges flow from the PM electrode to the ground through an external load, thereby generating a positive current pulse. After the completed separation of the SR, the device is in the electrostatic equilibrium state. Subsequently, the SR-PI triboelectric pair moves toward each other, and the charges on the PM electrode are driven to flow back to the ground via the load; thus, an opposite current is obtained. Thus, during the separating and approaching cycles of the triboelectric pair, electrons are induced to flow between the electrode and ground to form currents. **b** Illustration of the working mechanism of EMG. During these separating and approaching cycles, the electromagnetic nanogenerator also induces current pulses due to electromagnetic induction.

Figure 2 schematically illustrates the working mechanism of the EHTE, which is a hybrid mechanism based on the coupling of triboelectric and electromagnetic effects. Here, the working mechanism of the hybrid microenergy harvester was described in two parts of the triboelectric nanogenerator and the electromagnetic nanogenerator, as shown in Fig. 2a, b, respectively. Initially, the triboelectric nanogenerator is in an electrostatic neutral state. When the external force placed the silicone rubber–polyimide (SR-PI) triboelectric pair in close contact, equivalent numbers of triboelectric charges with opposite polarities were generated at the interface of the triboelectric pair due to the triboelectrification effect. According to the triboelectric series, polyimide is much more triboelectrically positive than silicone rubber. Therefore, the silicone rubber surface is negatively charged, and the polyimide surface is positively charged. When the external force releases, resulting in the silicone rubber leaving the polyimide surface, the internal electrical potential is established. The unbalanced negative charges cause the accumulation of positive charges on the permanent magnet electrode due to electrostatic induction. As a result, the accumulated charges flow from the PM electrode to the ground through an external load; and a current pulse is generated. After the completed separation of the silicone rubber and polyimide, the device is in the electrostatic equilibrium state. As silicone rubber moves toward the polyimide again, the charges on the permanent magnet electrode are driven to flow back to the ground via the load, and an opposite current is obtained. Therefore, during the separating and approaching cycles of the triboelectric pair, electrons are induced to flow between the PM electrode and ground to form currents. Meanwhile, the electromagnetic nanogenerator generates currents during the removal and approaching cycles between the PM and the FPCB coil due to electromagnetic induction. When the PM moves away from the coil, the magnetic flux crossing the FPCB coil decreases, resulting in the induced current flow in the FPCB coil. In contrast, as the PM approaches the coil, the magnetic flux crossing the coil increases, thereby inducing an opposite current in the FPCB coil. As a result, the hybrid microenergy harvester can convert mechanical input into triboelectric and electromagnetic outputs simultaneously according to the hybrid mechanism of triboelectrification and electromagnetism.

#### Triboelectric and electromagnetic outputs

**Fig. 3:**
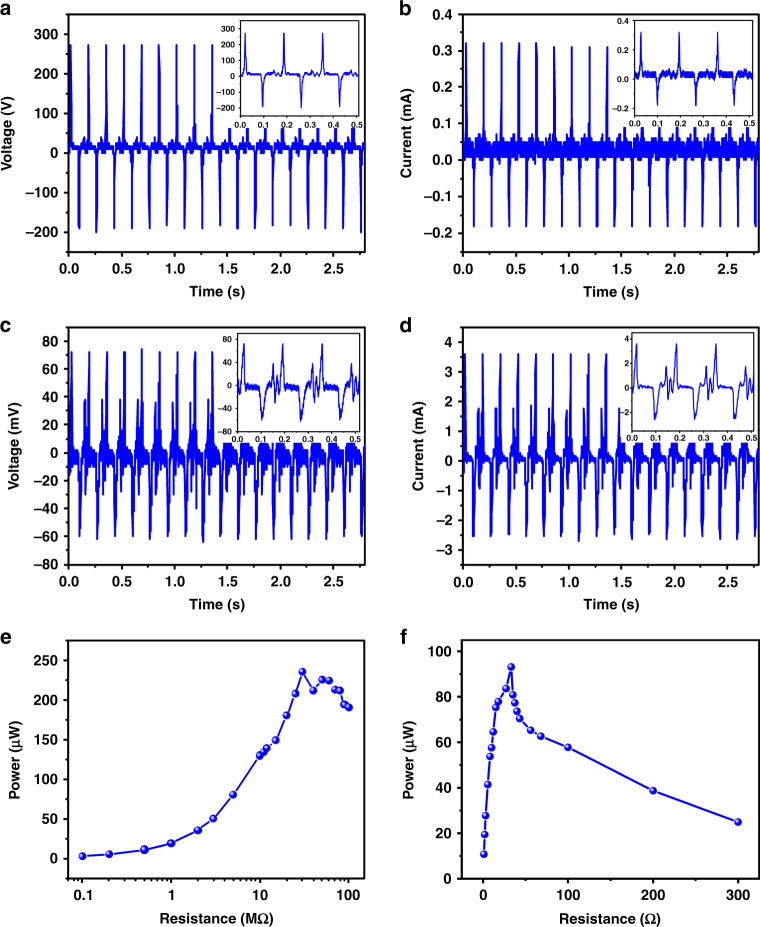
Triboelectric and electromagnetic outputs of the elastic hybrid triboelectric–electromagnetic microenergy harvester (EHTE). A vibration platform was used to supply controllable forces with designed frequencies. In this case, the frequency of the external force was 6 Hz. **a** Output voltage and **b** current of the triboelectric nanogenerator at 280 V and 0.32 mA, respectively. **c** Output voltage and **d** current of the electromagnetic nanogenerator at 72 mV and 3.6 mA, respectively. The results indicate that the triboelectric nanogenerator had a relatively high voltage output but a low current output, and the electromagnetic nanogenerator had a relatively high current output but a low voltage output, demonstrating the feasibility of the device to realize output complementation. **e** The output power on the loading resistors at various values from 0.1 to 100 MΩ connected to the triboelectric nanogenerator in parallel achieved a maximum value of 235.2 μW when the load resistance was 30 MΩ. **f** The output power on the loading resistors in values from 1 to 300 Ω connected to the electromagnetic nanogenerator in series achieved a maximum value of 93.1 μW when the load resistance was 45 Ω.

To provide a stable and controllable compression force with a designable frequency and strength for the hybrid microenergy harvester, a vibration platform consisting of a signal generating system, an amplifier and a shaker was used to help measure the electrical outputs of the hybrid microenergy harvester, as shown in supplementary Video S1. Figure 3 shows the electrical outputs of the individual parts of the proposed hybrid microenergy harvester (i.e., the triboelectric nanogenerator and the electromagnetic nanogenerator) under continuous forces (~35.4 N) with a fixed frequency of 6 Hz from the vibration platform. The peak output voltage and the peak output current of the triboelectric nanogenerator were ~280 V and 0.32 mA, as depicted in Fig. 3a, b, respectively. Additionally, the peak output voltage and peak output current of the electromagnetic nanogenerator were ~72 mV and 3.6 mA, respectively, as shown in Fig. 3c, d, respectively. These results indicated that the triboelectric and electromagnetic outputs of the hybrid microenergy harvester were basically consistent with the inherent output properties of TENGs and EMGs; i.e., the TENG of EHTE possessed a relatively high output voltage but low output current, and the EMG of EHTE had a relatively high output current but low output voltage. Thus, we used the triboelectric voltage and the electromagnetic current as the main parameters to further evaluate the electrical output characteristics of the proposed EHTE.

The output power was evaluated by connecting the individual parts of EHTE with external loading resistors. Herein, we connected a triboelectric nanogenerator with external loading resistors with values of 1 to 100 MΩ in parallel to measure the output peak voltage of the resistor and thereby its instantaneous output power. The output peak power of the triboelectric nanogenerator is shown in Fig. 3e. The triboelectric nanogenerator had a high internal resistance of ~30 MΩ, at which the maximum output peak power reached 235.2 μW. Meanwhile, the output power of the electromagnetic nanogenerator was obtained by connecting the electromagnetic nanogenerator with loading resistors with various values of 1 to 300 Ω in series. The internal resistance of the electromagnetic nanogenerator was estimated to be ~45 Ω, at which the output peak power was 93.1 μW, as shown in Fig. 3f. The area of the device was 28 × 28 mm; thus, the instantaneous power density of the triboelectric nanogenerator and the electromagnetic nanogenerator were calculated as 30.0 and 11.9 μW/cm^2^, respectively. Furthermore, fatigue tests of the EHTE were performed, as shown in Fig. S3 of the Supplementary File. After 10,800 working cycles, both the triboelectric nanogenerator and the electromagnetic nanogenerator maintained remarkable electrical outputs. The electromagnetic nanogenerator almost retained the initial output, and the 20% output decline of the triboelectric nanogenerator could be explained by the abrasion of the silicone rubber triboelectric layer after thousands of working cycles.

Notably, the electrical output presented in Fig. 3 is the output of the hybrid microenergy harvester using a ferroelastomer substrate with a 50% proportion of FeSiAl particles, which is the optimal proportion to obtain a higher output. Herein, the effect of the proportion of FeSiAl particles in the FeSiAl/SR ferroelastomeric substrate on the triboelectric and electromagnetic outputs was studied, and the triboelectric peak-to-peak output voltage and the electromagnetic peak-to-peak output current of EHTE with a ferroelastomeric substrate using varying concentrations of FeSiAl particles in silicone rubber are presented in Fig. S4. As the proportion of the FeSiAl particles increased from 0 to 50 wt%, the triboelectric peak-to-peak voltage of the device gradually increased from 368 V to 488 V, and the electromagnetic peak-to-peak current increased from 4.16 mA to 6.16 mA. As the FeSiAl proportion further increased to 75 wt%, the triboelectric peak-to-peak voltage increased to 544 V, but the electromagnetic peak-to-peak current declined to 5.6 mA. As a result, we chose the ferroelastomer with a 50 wt% proportion of FeSiAl particles as the ferroelastomeric substrate. Compared with the pure silicone rubber substrate-based hybrid microenergy harvester, the triboelectric peak-to-peak output voltage and the electromagnetic peak-to-peak output current of the FeSiAl/silicone rubber (50 wt% FeSiAl) substrate-based device increased by ~32.6% and 48.1%, respectively, indicating the importance of the ferroelastomeric substrate in the enhancement of the electrical output of the devices.

#### Effect of the excitation frequency on triboelectric and electromagnetic outputs

**Fig. 4:**
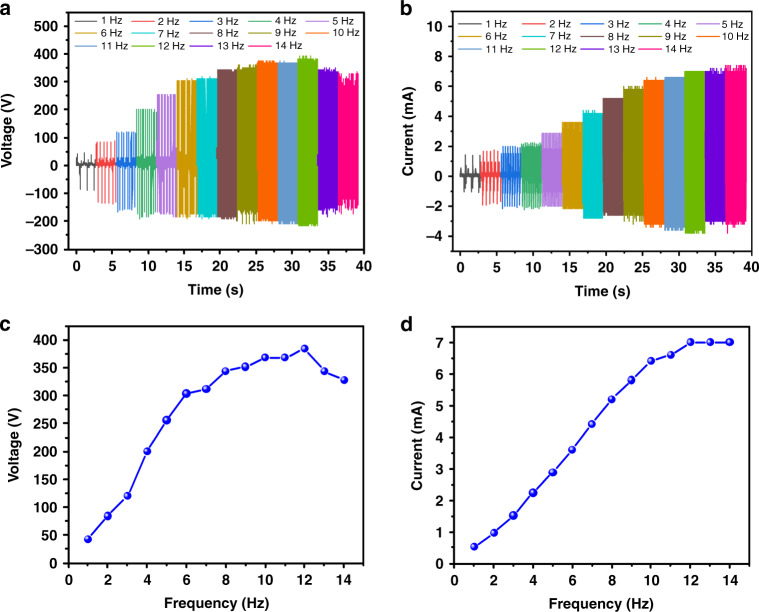
Effect of frequency on triboelectric and electromagnetic outputs. **a** Triboelectric voltage output and **b** electromagnetic current output at different frequencies in the range of 1 to 14 Hz. **c** Triboelectric voltage output and **d** electromagnetic current output versus the frequency of the external force. When the frequency of the external excitation increased from 1 to 12 Hz, the triboelectric voltage output gradually increased from 44 to 392 V, and the electromagnetic current output increased from 0.52 to 7.00 mA. However, due to the under-released effect of the device working in a relatively high frequency range, the triboelectric voltage output declined to 336 V, and the electromagnetic current output remained at almost 7.00 mA, but its negative peak values declined when the external frequency further increased to 14 Hz. The results show that the triboelectric and electromagnetic outputs were highly related to the frequency of the external force. Moreover, the linear correlation between the electromagnetic current outputs corresponding to frequencies in the range of 1 to 10 Hz provides the opportunity for hybrid microenergy harvesters for active frequency sensing.

The reliance of triboelectric and electromagnetic outputs on the frequency of the external excitation was also studied, as illustrated in Fig. 4. The triboelectric voltage output and the electromagnetic current output of the hybrid microenergy harvester at different frequencies are shown in Fig. 4a, b, respectively. When the frequency increased from 1 to 12 Hz with an increasing step of 1 Hz, the triboelectric voltage gradually increased from 44 to 392 V, and the electromagnetic current increased from 0.52 to 7.00 mA. However, with a frequency increase greater than 12 Hz (i.e., 13 and 14 Hz), the triboelectric voltage output declined to 212 V, and the electromagnetic current outputs were maintained at 7.00 mA, but its negative peak current gradually declined. The declining trend could be explained by the under-recover effect of the hybrid microenergy harvester since the excessively short time span of the applied force did not allow it to recover to the original status completely. It is noted that the values of the triboelectric and electromagnetic outputs were the average values of the positive peaks of the waveforms. In this case, the correlation curves of the triboelectric voltage output and the electromagnetic current output to the frequency of the external excitation are presented in Fig. 4c, d, respectively. The electromagnetic current output was almost linear to the applied frequency of the external excitation in the range of 1–10 Hz, demonstrating the potential of the EHTE for active frequency sensing.

#### EHTE serves as a power source for microelectronics powering

**Fig. 5:**
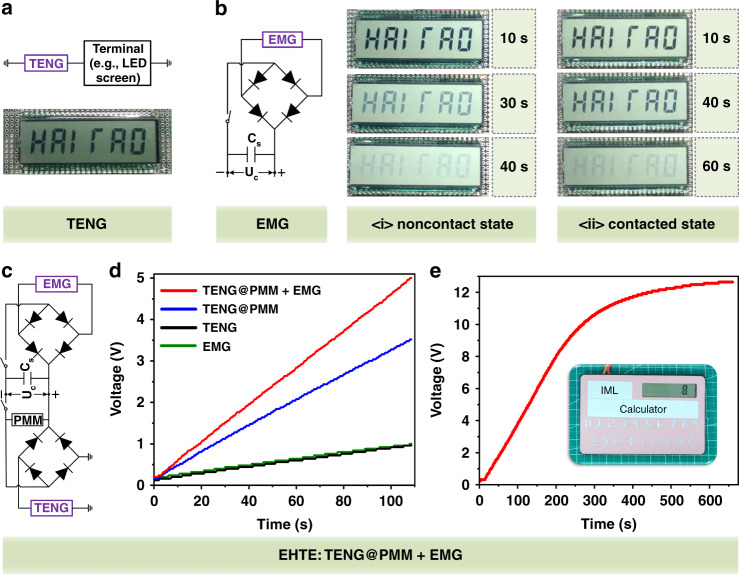
The EHTE serves as a mechanical microenergy harvester to directly/indirectly drive microelectronics. **a** An LED screen was directly lit by a triboelectric nanogenerator (TENG) only. **b** The LED screen was indirectly lit by an electromagnetic nanogenerator (EMG) with the help of a rectifier circuit and an energy storage capacitor. Powering ability of the EMG working in the <i> noncontact state and <ii> contacted state. **c**–**e** Illustration of the EHTE to drive a commercial calculator. **c** Schematic diagram of the charging circuit of the hybrid microenergy harvester. **d** Comparison of the charging behaviors of the EMG, TENG, TENG with a power management circuit (TENG@PMM), and EHTE (TENG@PMM + EMG). Herein, a 10 μF-capacitor was charged to 1.00, 0.96, 3.52, and 5.00 V within 110 s by EMG, TENG, TENG@PMM, and TENG@ PMM + EMG, respectively. With the help of the PMM and the combination of triboelectric and electromagnetic outputs, the charging ability of EHTE was clearly enhanced. **e** The 10 μF-capacitor was charged by EHTE and successfully drove a commercial calculator. The inset view shows the commercial calculator in the driven state.

The remarkable electrical outputs of the elastic hybrid triboelectric–electromagnetic microenergy harvester provide the feasibility of the device to be used as a power source for microelectronics powering. As illustrated in Fig. 5a, the triboelectric nanogenerator of the hybrid microenergy harvester could be used to directly drive an LED screen, owing to its high voltage property. With the help of a rectifier circuit and an energy storage capacitor, the electromagnetic nanogenerator could successfully light up the LED screen in both the noncontact working state and the contacted working state, as shown in Fig. 5b<i>, <ii>, respectively. It is noted that the working state of the electromagnetic nanogenerator was controlled by setting the amplitude of the external excitation from the vibration platform. More details about the effect of the amplitude of the external excitation on electromagnetic output are shown in Fig. S5 of the supplementary file. Herein, a 1 μF-capacitor was charged for 360 s by the electromagnetic nanogenerator of the EHTE working in the noncontact state and contacted state, and then the stored energy was used to power the LED screen. The former can make the LED screen display normally for 25 s and then gradually darken and disappear after 40 s. The latter can make the display work normally for ~40 s and then gradually darken and disappear after 62 s.

Moreover, to enhance the power delivery efficiency of the TENG, a power management circuit (PMM) developed in a previous study^9^ by our group was used to improve the tremendous impedance mismatch between the triboelectric nanogenerator and the energy storage unit. Figure 5c schematically illustrates the charging circuit of the hybrid microenergy harvester, and more detail about the PMM is schematically illustrated in Fig. S6. Herein, the charging ability of the electromagnetic nanogenerator (EMG), the triboelectric nanogenerator only (TENG), the TENG with a power management circuit (TENG@PMM), and the hybrid nanogenerator (TENG@PMM + EMG) to an external capacitor with a value of 10 μF was studied. As shown in Fig. 5d, within a charging time of 110 seconds, the external capacitor can be charged to 0.96 and 1.00 V by the TENG only (i.e., the black curve) and EMG only (i.e., the green curve), respectively. With the help of the PMM, the output voltage of the charged capacitor increased to 3.52 V under the same conditions by TENG@PMM (i.e., the blue curve). The output voltage of the charged capacitor was further enhanced to 5 V by TENG@PMM + EMG (i.e., the red curve) resulting from the hybrid effect of the triboelectric and electromagnetic outputs, which indicates that the charging ability of the proposed hybrid microenergy harvester is remarkable. As shown in Fig. 5e, the developed EHTE could take 255 s to charge the 10 μF-capacitor to 10 V, and it was successfully demonstrated to drive a commercial calculator, as shown in the inset view of Fig. 5e, which reveals the feasibility of the EHTE for wearable microelectronics powering.

### Inductive sensing based on the eddy current effect

**Fig. 6:**
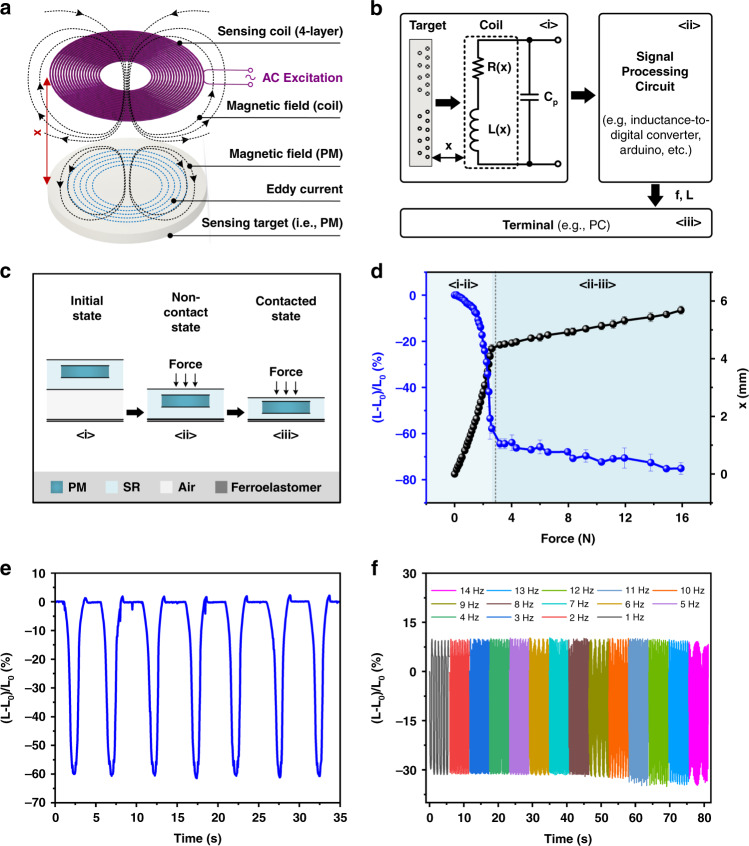
EHTE serves as an inductive sensor for passive pressure sensing. **a** Sensing mechanism of the inductive sensor. When the sensing coil was excited by an AC current, the coil generated an alternative magnetic field, inducing eddy currents in the nearby conductive sensing target (i.e., PM) due to the eddy current effect. The induced eddy currents on the PM surface simultaneously generated a magnetic field opposite to the magnetic field generated by the coil. The magnetic field coupling between the coil and the PM results in a decrease in the magnetic flux in the coil and thus its effective inductance. When the external force was applied to make the PM close to the coil, the induced eddy currents increased, and thus the effective inductance of the coil decreased. **b** Schematic illustration of the inductance measurement system, including <i> the EHTE at which the coil was connected with an external capacitor in parallel to form an LC oscillator for inductance measurement, <ii> a signal processing circuit mainly consisting of an inductance-to-digital converter and an Arduino microcontroller to drive the LC network and measure its oscillating frequency, thereby the effective inductance of the coil, and <iii> a PC terminal to receive and display the measured inductance of the coil. **c** Schematic diagram of one compression process of the inductive sensor, including two stages of the <i-ii> noncontact stage (i.e., the compression of the air layer) and the <ii-iii> contacted stage (i.e., the compression of silicone rubber and ferroelastomer in the whole device). **d** Relative inductance change in the coil versus the external force and the device deformation versus the external force. This inductive sensor had a maximum sensitivity of 0.570 N^−1^. **e** Relative inductance change in the coil under seven continuous cycles of the working process. **f** Effect of the excitation frequency on the relative inductance change in the coil, indicating that the frequency of the external excitation had no obvious effect on the inductive sensing performance of the device.

Inspired by the configuration of the eddy current sensor composed of two essential parts of the sensing coil and conductive target, the assembly of the conductive permanent magnet (PM) and 4-layer FPCB coil in the electromagnetic nanogenerator of the EHTE demonstrates the feasibility of inductive sensing realized by the eddy current effect. Herein, the conductive PM is regarded as the sensing target of the inductive sensor, and the 4-layer FPCB coil is regarded as its sensing coil. Figure 6a schematically illustrates the sensing mechanism of the inductive sensor. When the sensing coil (i.e., 4-layer FPCB coil) is excited by an alternative current (AC), the coil generates an alternative magnetic field, which induces eddy currents in the nearby conductive sensing target (i.e., PM) due to the eddy current effect. The induced eddy currents on the surface of the sensing target generate a magnetic field in a direction opposite to the magnetic field generated by the sensing coil, which reduces the magnetic flux crossing the sensing coil and thereby the effective inductance of the sensing coil due to the magnetic field coupling between the sensing coil and target. In addition, the resistance of the sensing coil increases due to the energy dissipation of the eddy currents. The induced eddy currents are affected by the distance between the sensing target and coil. Therefore, when the compression force moves the sensing target (i.e., PM) toward the sensing coil (i.e., FPCB coil), the induced eddy currents increase so that the magnetic field coupling between the sensing coil and the sensing target increases and the effective inductance of the sensing coil decreases. Thus, the sensing performance of the inductive sensor could be qualitatively evaluated by the inductance response behavior of the FPCB coil to the external compression force.

Herein, an inductance measurement system was built to obtain the inductance changes in the sensing coil (i.e., FPCB coil), including the hybrid microenergy harvester at which the FPCB coil (the coil was equivalent to an LR series circuit) was connected with an external capacitor in parallel to form an LC oscillator for inductance measurements, a signal processing circuit and a PC terminal, as shown in Fig. 6b. The signal processing circuit was mainly comprised of an inductance-to-digital converter and an Arduino microcontroller, and more details about the signal processing circuit are given in Fig. S7 of the supplementary file. The inductance-to-digital converter was used to drive the LC network and measure its oscillating frequency and thereby the effective inductance of the FPCB coil, according to the correlation of the oscillating frequency and the inductance of the LC network, i.e., $f = \frac{1}{{2\pi \sqrt {{\mathrm{LC}}} }}$, where *f* is the oscillating frequency of the LC network, *L* is the inductance of the FPCB coil, and *C* is the external capacitance. The FPCB coil on the hybrid microenergy harvester had an inductance value of 78.84 μH; therefore, we chose a 330 pF capacitor to form the LC oscillator with an oscillating frequency of ~0.98 MHz, which was in the measurable frequency range of the inductance-to-digital converter (1 kHz ~ 10 MHz). When the FPCB coil was driven by an AC current at the oscillating frequency of the LC network, the inductance output was rendered by the inductance-to-digital converter and sent to the Arduino controller via the I2C protocol, which was finally displayed on a PC terminal in real-time.

Figure 6c schematically illustrates the working process of the hybrid microenergy harvester serving as an inductive sensor, which includes two stages: the (i–ii) noncontact stage and the (ii–iii) contacted stage. This behavior was mainly caused by the structural characteristics of the hybrid deformation layer of the hybrid microenergy harvester so that the air layer of the deformation layer was first deformed (Fig. 6c(i–ii)) and then deformed in the elastomeric layers in the whole device (Fig. 6c(ii–iii)), including the silicone rubber layer and the ferroelastomeric layer. Herein, a push-and-pull tester was used to provide the external force to the device and display the device deformation simultaneously. Figure 6d illustrates the inductance response of the FPCB coil versus the external force (i.e., blue curve) and the whole deformation of the inductive sensor versus the external force (i.e., black curve). The initial inductance of the integrated FPCB coil was ~95 μH. When an increasing compression force was applied, the PM gradually moved close to the FPCB coil, and the inductance of the FPCB coil declined, with a maximum sensitivity of 0.570 N^−1^ in the noncontacted stage (i.e., (i–ii)) and a sensitivity of 0.008 N^−1^ in the contacted stage (i.e., (i–ii)), and a degree of hysteresis (DH) of 18.2% is shown in Fig. S8 of the supplementary file. Moreover, the oscillating frequency in the LC network versus the external force is given in Fig. S9 of the supplementary file. Figure 6e shows the inductance response behavior of the coil under seven continuous cycles of the compression–release process, which indicated a dynamic stability of the device. To study the anti-fatigue ability of the inductive sensor, we also studied the inductance outputs of the device under ten thousand cyclic compression–release cycles using the vibration platform to provide long-term stable and controllable forces. Figure S10 shows that the inductive sensor can maintain stable inductance changes under 10400 working cycles and indicates that the repeatability of the sensor is outstanding. The short-term and long-term stability of the sensor was also studied in this case. After standing for 1 day and 9 months, only 5.2% and 3.2% fluctuation existed, respectively, as shown in Fig. S11, showing outstanding short-term and long-term stability. Moreover, the effect of the frequency of the external force on the inductance changes was investigated in this section. As depicted in Fig. 6f, the relative inductance change in the FPCB coil can be maintained at 31.3% as the frequency of the external force increases from 1 to 8 Hz. When the frequency further increased from 8 to 14 Hz, an increasing trend of the relative inductance change was presented. This can be explained by the large inertia force from the shaker of the vibration platform under a relatively high frequency, resulting in an enlarged deformation of the device. The results indicated that the inductance changes in the FPCB coil have no effect on the frequency of the external force. It is worth mentioning that the inductance increment exists for each working cycle of Fig. 6e, f, which is due to the high viscoelasticity of the silicone rubber encapsulation layer. As a result, the device cannot be separated from the head supplying the external force immediately when the device recovers to the initial state, as shown in Fig. 6c(i), but it moves upward with the head for a moment, resulting in an increase in the distance between the sensing target and the sensing coil, thereby increasing the effective inductance of the sensing coil. In addition, we studied the inductive sensing performance of the device with different materials. The details about them are given in Fig. S12 of the supplementary file. Herein, we tested the relative inductance changes in the device to the external press from different material-based objects, including the press of a glass beaker with 20 g water, the vibration of the aluminum block, the finger press, and the press of plastics, with average relative inductance changes of 0.78%, 29.2%, 30.1%, and 60.6%, respectively.

## Conclusion

In summary, an elastic hybrid triboelectric–electromagnetic microenergy harvester (EHTE) was developed, which consisted of an FeSiAl/SR ferroelastomer substrate, a 4-layer FPCB coil, an SR-air hybrid deformation layer, and a PM and an SR encapsulation layer. This new device was demonstrated to successfully achieve the integration of hybrid microenergy and sensing in a single device for compact active microsystems. Based on the triboelectric–electromagnetic hybrid mechanism, the EHTE can generate triboelectric and electromagnetic outputs simultaneously from one mechanical input, with a triboelectric output voltage, current and power density of 280 V, 0.32 mA, and 30.0 μW/cm^2^, respectively, and electromagnetic output voltage, current and power density of 72 mV, 3.6 mA, and 11.9 μW/cm^2^, respectively. The triboelectric and electromagnetic outputs were demonstrated to power microelectronics, such as LED display screens. Moreover, the hybrid output exhibited an enhanced output power, which is large enough to power wearable microelectronics such as commercial calculators. For hybrid sensing, EHTE was proven to achieve both active frequency sensing and passive inductive sensing based on electromagnetic induction. Specifically, the electromagnetic nanogenerator was verified to linearly sense the frequency of the external excitation in the frequency range of 1~10 Hz. Moreover, based on the eddy current effect, the proposed EHTE can be used as an inductive sensor for passive sensing according to the quantitative relation between the inductance change in the sensing coil (i.e., FPCB coil) and the external force. Therefore, the developed EHTE successfully achieved the integration of hybrid microenergy, active frequency sensing and passive inductive sensing in a compact form, which provides promising potential for wearable electronic and active microsystem applications.

## Methods

### Fabrication of the developed EHTE

**Fig. 7:**
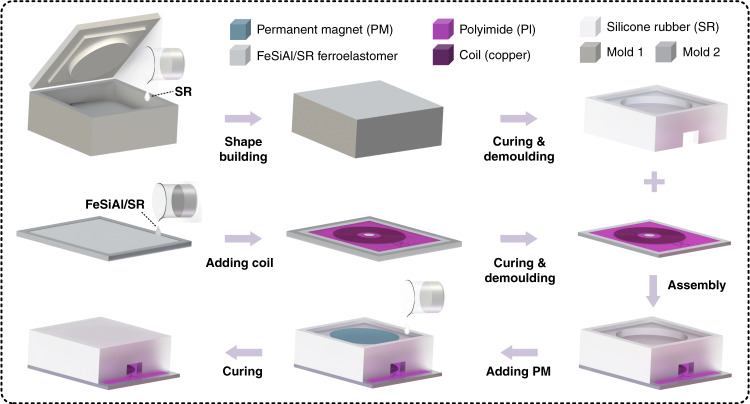
Schematic illustration of the fabrication process flow chart of the developed elastic hybrid triboelectric–electromagnetic microenergy harvester (EHTE). First, the liquid ferroelastomer was prepared by mixing FeSiAl particles and liquid silicone rubber (SR) at a weight ratio of 1:1. Then, the prepared liquid ferroelastomer was dispensed atop a 3D printing mold with a cuboid pattern (i.e., mold 1), followed by vacuum defoaming to remove bubbles in the liquid ferroelastomer and at the interface between mold and liquid ferroelastomer. Second, a 4-layer FPCB coil was gently placed on the surface of the liquid ferroelastomer. After vacuum defoaming and room temperature curing successively, the FPCB coil was successfully embedded onto the surface of the FeSiAl/SR ferroelastomeric substrate. A ferroelastomeric substrate with an FPCB coil was obtained by peeling it off mold 1. Third, the SR deformation layer replicated from mold 2 was bonded to the obtained FeSiAl/SR ferroelastomeric substrate by dipping a small amount of liquid SR at their interface. After curing the SR at room temperature, a permanent magnet (PM) was placed in the top round groove of the SR deformation layer and encapsulated by liquid SR. Finally, EHTE was obtained by curing the liquid SR at room temperature. It is noted that the used SR was prepared by mixing Part A and Part B solutions of Ecoflex 00-30 rubber at a weight ratio of 1:1.

Figure 7 schematically illustrates the fabrication process flow of the developed elastic hybrid triboelectric–electromagnetic microenergy harvester (EHTE), including six main steps. First, the liquid ferroelastomer was prepared by mixing FeSiAl particles (diameter: 4 –20 μm) and liquid silicone rubber (SR, Ecoflex 00-30) at a weight ratio of 1:1. Then, the liquid ferroelastomer (FeSiAl/SR) was dispersed atop a prepared 3D printing mold with a cuboid pattern (i.e., mold 1), followed by vacuum defoaming to remove bubbles in the liquid ferroelastomer and at the interface between mold 1 and the liquid ferroelastomer. Second, a 4-layer FPCB coil was gently placed on the surface of the obtained liquid ferroelastomer. After vacuum defoaming and curing at room temperature successively, the FPCB coil can be successfully embedded on the surface of the FeSiAl/SR ferroelastomeric substrate. The ferroelastomeric substrate embedded with the FPCB coil was obtained by peeling it off mold 1. Third, some liquid silicone rubber was successively dropped into the other 3D-printed mold (i.e., mold 2), followed by vacuum defoaming and room temperature curing. The designed silicone rubber–air hybrid deformation layer was obtained by replicating it from mold 2. Fourth, the obtained deformation layer was bonded with the ferroelastomeric substrate with an embedded FPCB coil by dispersing a thin layer of liquid silicone rubber at their interface. Fifth, after curing the liquid silicone rubber at room temperature, a conductive permanent magnet (PM, with a thickness of 2 mm and diameter of 20 mm) was placed in the top round groove of the deformation layer and was encapsulated by liquid silicone rubber. Finally, the hybrid microenergy harvester was fabricated by curing the liquid silicone rubber at room temperature. It is noted that the liquid silicone rubber used in the whole process was prepared by mixing Part A and Part B of the silicone rubber solution (Ecoflex 00-30) at a weight ratio of 1:1.

### Tests and measurements

The surface morphologies of the FeSiAl/SR ferroelastomer were characterized using scanning electron microscopy (SEM, JSM-6490LV, JEOL Ltd.). The magnetic hysteresis loops of the FeSiAl/SR ferroelastomer were tested by a vibrating sample magnetometer (8600 Series VSM, Lake Shore CRYOTRONICS). A vibration platform consisting of a signal generating system (33250A, Agilent), an amplifier and a shaker was used to provide a stable and controllable force with designable frequency to the device. The triboelectric voltage outputs were measured using a digital oscilloscope (DS2302A, RIOGL) with a 100 MΩ probe, and the triboelectric current outputs were measured by a low-noise current preamplifier (SR570, Stanford Research Systems) and a digital oscilloscope (DS2302A, RIOGL) with a 100 MΩ probe. The electromagnetic outputs were measured using an electrometer (6514, KEITHLEY) and a digital oscilloscope (DS2302A, RIOGL) with a 1 MΩ probe. The charging curves of the device were tested by a digital oscilloscope (DS2302A, RIOGL) with a 100 MΩ probe. The external force applied by the vibration platform was tested by a commercial force sensor connected to a force gauge (HP-160, HANDPI).

## Supplementary information

Supplementary file

Supplementary Information video

## References

[1] Serror M, Hack S, Henze M, Schuba M, Wehrle K (2021). Challenges and opportunities in securing the industrial internet of things. IEEE Trans. Ind. Inf..

[2] Li Z, Liu Y, Liu A, Wang S, Liu H (2020). Minimizing convergecast time and energy consumption in green internet of things. IEEE T. Emerg. Top. Com..

[3] Claro MS, Stroppa DG, da Silva ECF, Quivy AA (2020). Strong photovoltaic effect in high-density InAlAs and InAs/InAlAsquantum-dot in frared photodetectors. Sens. Actuators A.

[4] He Q (2020). Surface passivation of perovskite thin films by phosphonium halides for efficient and stable solar cells. J. Mater. Chem. A.

[5] Wei X, Peng Y, Jing G, Simon T, Cui T (2021). High-performance perovskite solar cells fabricated by a hybrid physical-chemical vapor deposition. J. Sol. Energy Eng..

[6] Bell LE (2008). Cooling, heating, generating power, and recovering waste. Heat. Thermoelectr. Syst. Sci..

[7] Liu L (2017). A photovoltaic self-powered gas sensor based on a single-walled carbon nanotube/Si heterojunction. Nanoscale.

[8] Wen DL (2020). Wearable multi-sensing double-chain thermoelectric generator. Microsyst. Nanoeng..

[9] Maharjan P (2019). High-performance cycloid inspired wearable electromagnetic energy, harvester for scavenging human motion energy. Appl. Energy.

[10] Beeby SP (2007). A micro electromagnetic generator for vibration energy harvesting. J. Micromech. Microeng..

[11] Annapureddy V (2017). Magnetic energy harvesting with magnetoelectrics: an emerging technology for self-powered autonomous systems. Sustain. Energy Fuels.

[12] Hu D (2019). Strategies to achieve high performance piezoelectric nanogenerators. Nano Energy.

[13] Lee EJ (2018). High-performance piezoelectric nanogenerators based on chemically-reinforced composites. Energy Environ. Sci..

[14] Zhou D (2020). A piezoelectric nanogenerator promotes highly stretchable and self-chargeable supercapacitors. Mater. Horiz..

[15] Fan FR, Tian ZQ, Wang ZL (2012). Flexible triboelectric generator. Nano Energy.

[16] Long L (2021). High performance floating self-excited sliding triboelectric nanogenerator for micro mechanical energy harvesting. Nat. Commun..

[17] Hu Y, Luo A, Wang J, Wang F (2019). Voltage regulation and power management for wireless flow sensor node self-powered by energy harvester with enhanced reliability. IEEE Access.

[18] Wang H, Han M, Song Y, Zhang H (2021). Design, manufacturing and applications of wearable triboelectric nanogenerators. Nano Energy.

[19] Fu X (2021). Breeze-wind-energy-powered autonomous wireless anemometer based on rolling contact-electrification. ACS Energy Lett..

[20] Liu G, Chen T, Xu J, Wang K (2018). Blue energy harvesting on nanostructured carbon materials. J. Mater. Chem. A.

[21] Xu C, Song Y, Han M, Zhang H (2021). Portable and wearable self-powered systems based on emerging energy harvesting technology. Microsyst. Nanoeng..

[22] Son EJ, Kim JH, Kim K, Park CB (2016). Quinone and its derivatives for energy harvesting and storage materials. J. Mater. Chem. A.

[23] Miao L (2021). 3D temporary-magnetized soft robotic structures for enhanced energy harvesting. Adv. Mater..

[24] Zhang XS (2018). All-in-one self-powered flexible microsystems based on triboelectric nanogenerators. Nano Energy.

[25] Wang ZL (2013). Triboelectric nanogenerators as new energy technology for self-powered systems and as active mechanical and chemical sensors. ACS Nano.

[26] Tang W (2015). Liquid-metal electrode for high-performance triboelectric nanogenerator at an instantaneous energy conversion efficiency of 70.6%. Adv. Funct. Mater..

[27] Wen DL (2019). Printed silk-fibroin-based triboelectric nanogenerators for multi-functional wearable sensing. Nano Energy.

[28] Xu C (2022). Raindrop energy-powered autonomous wireless hyetometer based on liquid-solid contact electrification. Microsyst. Nanoeng..

[29] Liu Z, Zhao Z, Zeng X, Fu X, Hu Y (2019). Expandable microsphere-based triboelectric nanogenerators as ultrasensitive pressure sensors for respiratory and pulse monitoring. Nano Energy.

[30] Song Y, Mukasa D, Zhang H, Gao W (2021). Self-powered wearable biosensors. Acc. Mater. Res.

[31] Deng HT (2021). Super-stretchable multi-sensing triboelectric nanogenerator based on liquid conductive composite. Nano Energy.

[32] Ryu H, Yoon HJ, Kim SW (2019). Hybrid energy harvesters: toward sustainable energy harvesting. Adv. Mater..

[33] Sharov VA (2019). InP/Si heterostructure for high-current hybrid triboelectric/photovoltaic generation. ACS Appl. Energy Mater..

[34] Jung S (2020). 3D Cu ball-based hybrid triboelectric nanogenerator with non-fullerene organic photovoltaic cells for self-powering indoor electronics. Nano Energy.

[35] Su L (2016). Photoinduced enhancement of a triboelectric nanogenerator based on an organolead halide perovskite. J. Mater. Chem. C..

[36] Guo Y (2018). All-fiber hybrid piezoelectric-enhanced triboelectric nanogenerator for wearable gesture monitoring. Nano Energy.

[37] Li M (2019). All-in-one cellulose based hybrid tribo/piezoelectric nanogenerator. Nano Res.

[38] Chen X (2017). A wave-shaped hybrid piezoelectric and triboelectric nanogenerator based on P(VDF-TrFE) nanofibers. Nanoscale.

[39] Shao H (2018). Triboelectric-electromagnetic hybrid generator for harvesting blue. Energy Nano-Micro Lett..

[40] Han Q (2020). Hybrid triboelectric-electromagnetic generator for self-powered wind speed and direction detection. Sustain. Energy Technol. Assess..

[41] Chen YL, Liu D, Wang S, Li YF, Zhang XS (2019). Self-powered smart active RFID tag integrated with wearable hybrid nanogenerator. Nano Energy.

[42] Wan J (2020). A flexible hybridized electromagnetic-triboelectric nanogenerator and its application for 3D trajectory sensing. Nano Energy.

[43] Wang P (2018). Complementary electromagnetic-triboelectric active sensor for detecting multiple mechanical triggering. Adv. Funct. Mater..

[44] Wang S, Wang ZL, Yang Y (2016). A one-structure-based hybridized nanogenerator for scavenging mechanical and thermal energies by triboelectric-piezoelectric-pyroelectric effects. Adv. Mater..

[45] He J (2018). Triboelectric-piezoelectric-electromagnetic hybrid nanogenerator for high efficient vibration energy harvesting and self-powered wireless monitoring system. Nano Energy.

[46] Wang H, Feng Z (2013). Ultrastable and highly sensitive eddy current displacement sensor using self-temperature compensation. Sens. Actuators A.

[47] Wang H (2018). Robust and high-performance soft inductive tactile sensors based on the eddy-current effect. Sens. Actuators A.

