# Supplementary material for: Integrated hybrid sensing and microenergy for compact active microsystems
Source: Microsyst Nanoeng. 2022 Jun 6;8:61. doi: 10.1038/s41378-022-00393-z (PMC9170723; doi:10.1038/s41378-022-00393-z)
Supplement: Supplementary file 1 — Supplementary file [file 41378_2022_393_MOESM1_ESM.docx]

**Support Information**

Integrated hybrid sensing and microenergy for compact active microsystems

Hai-Tao Deng^1^, Zhi-Yong Wang^1^, Yi-Lin Wang^1^, Dan-Liang Wen^1^, Xiao-Sheng Zhang^1,*^

^1^School of Electronic Science and Engineering, University of Electronic Science and Technology of China, Chengdu 611731, China

*Corresponding Author: [zhangxs@uestc.edu.cn](mailto:zhangxs@uestc.edu.cn) (XS Zhang)

**
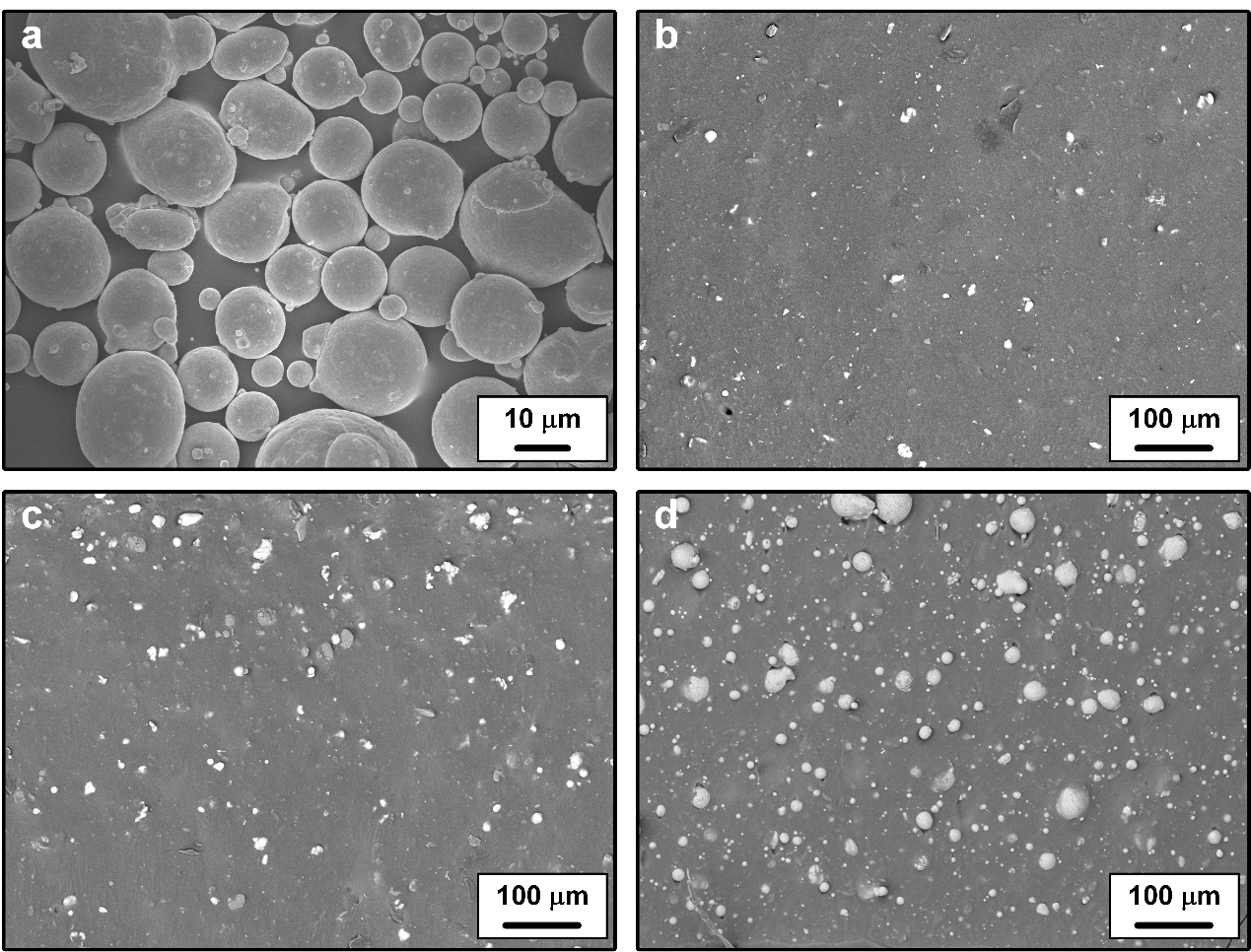
**

**Fig. S1** Surface morphologies of pure FeSiAl particles and FeSiAl/SR ferroelastomers with different proportion of FeSiAl particles. (a) Scanning electron microscope image of pure FeSiAl particles with non-uniform diameter of 4 μm~20 μm. (b-d) Backscatter electron microscope images of FeSiAl/SR ferroelastomer with (b) 25 wt% proportion of FeSiAl particles, (c) 50 wt% proportion of FeSiAl particles, and (d) 75 wt% proportion of FeSiAl particles.


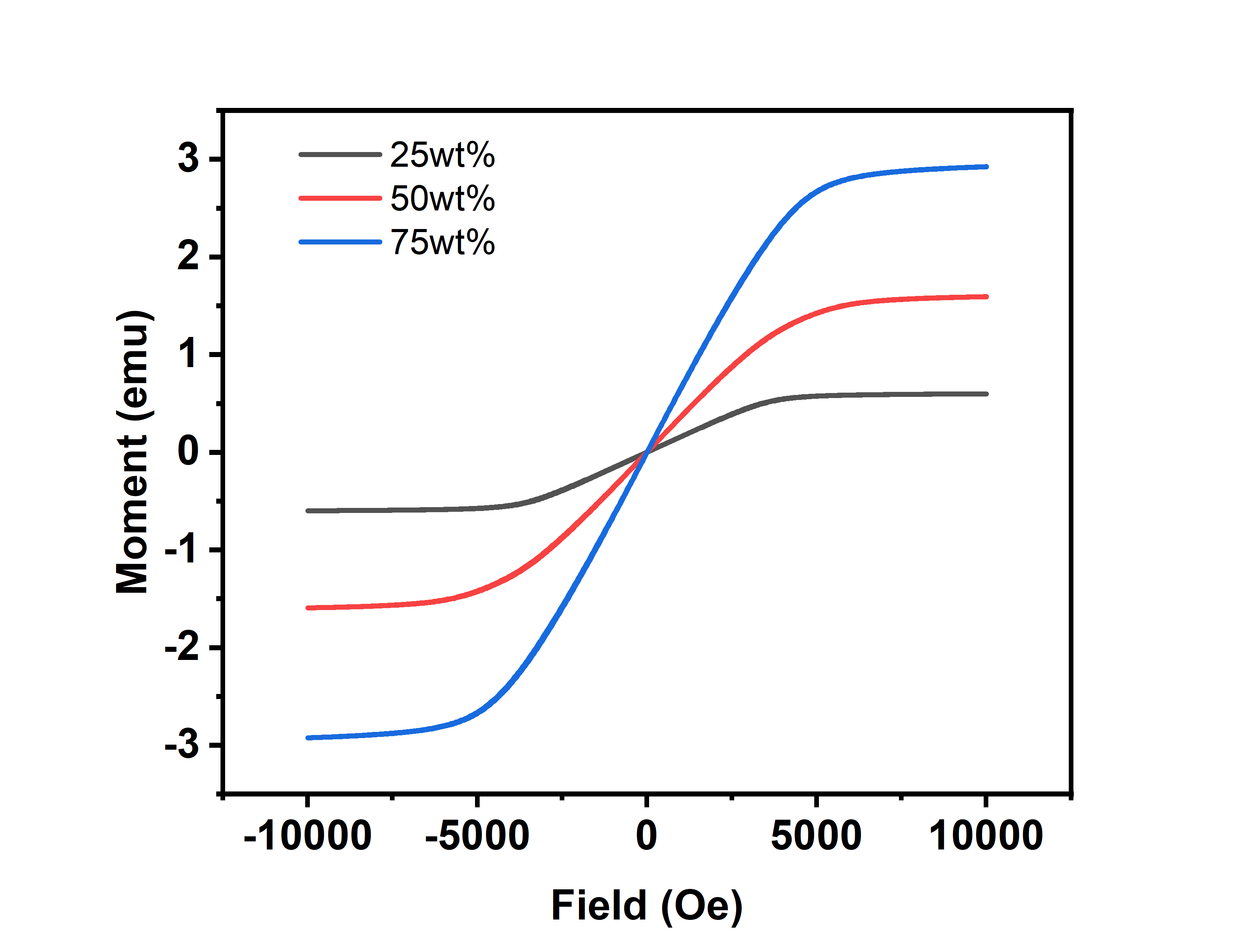


**Fig. S2** Magnetic hysteresis loops of FeSiAl/SR ferroelastomers with 25 wt%, 50 wt% and 75 wt% proportion of FeSiAl particles.


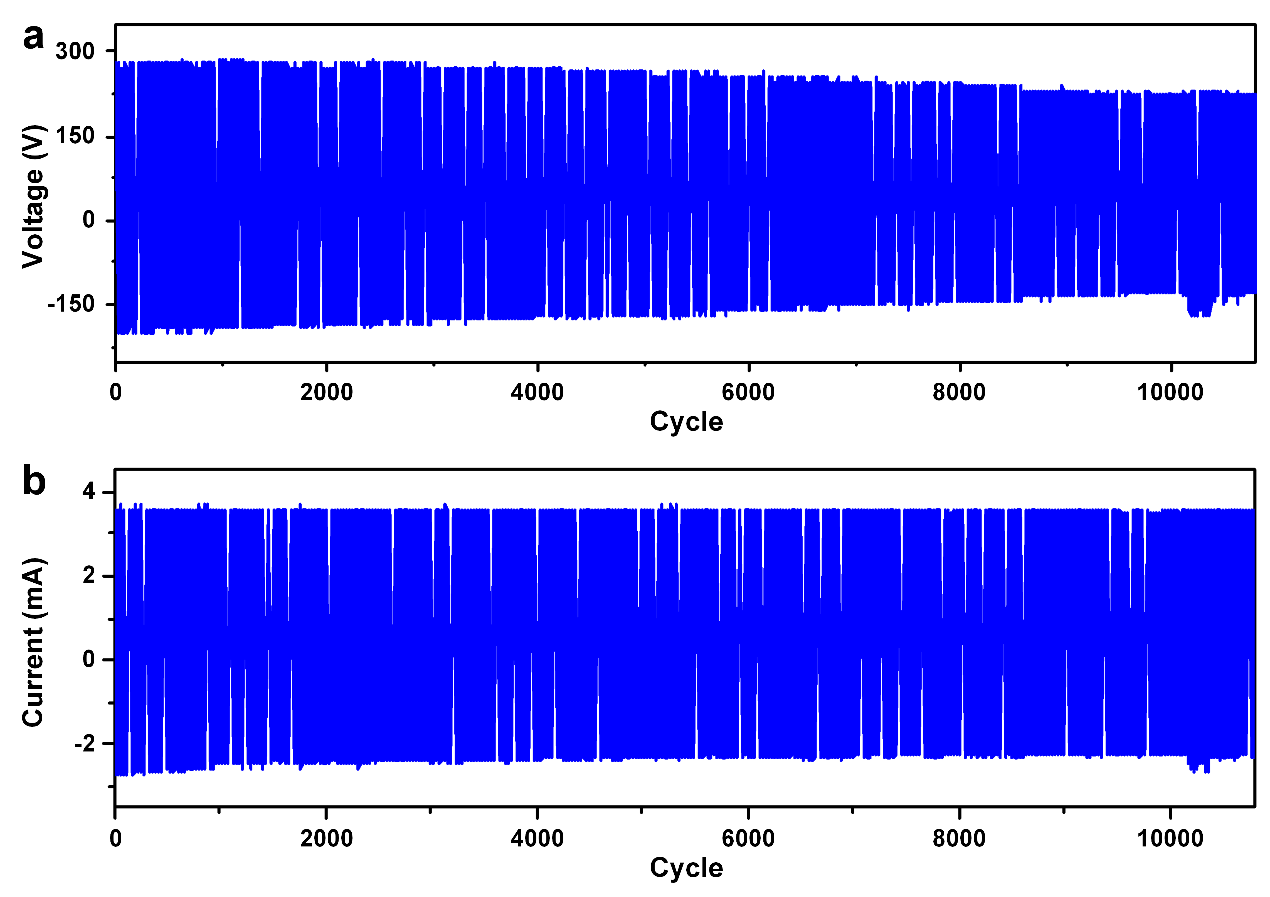


**Fig. S3** Fatigue tests of the EHTE serving as microenergy harvester. (a) Triboelectric voltage output and (b) electromagnetic current output of EHTE under 10800-cycle of the working process. The declined trend of the triboelectric voltage was caused by the abrasion of the silicone rubber triboelectric layer after thousands of working cycles.


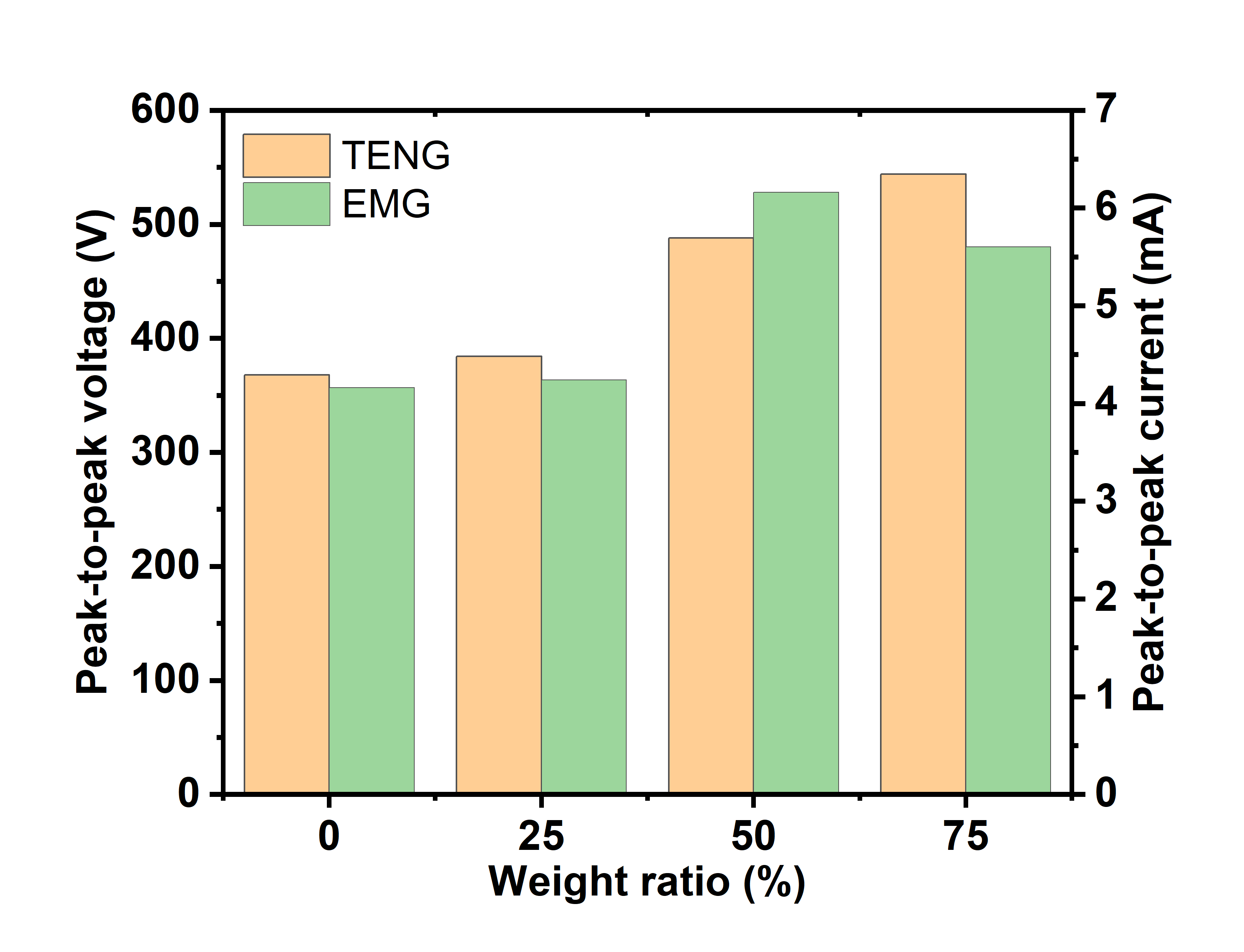


**Fig. S4** Triboelectric and electromagnetic outputs of the hybrid microenergy harvester using FeSiAl/SR ferroelastomers with 0 wt%, 25 wt%, 50 wt%, and 75 wt% proportion of FeSiAl particles. Considering that the larger proportion of magnetic particles, the higher hardness of the ferroelastomer, and the positive effect of the magnetic particles on the triboelectric and electromagnetic outputs, the FeSiAl proportion of the ferroelastomeric substrate was chose 50 wt%.


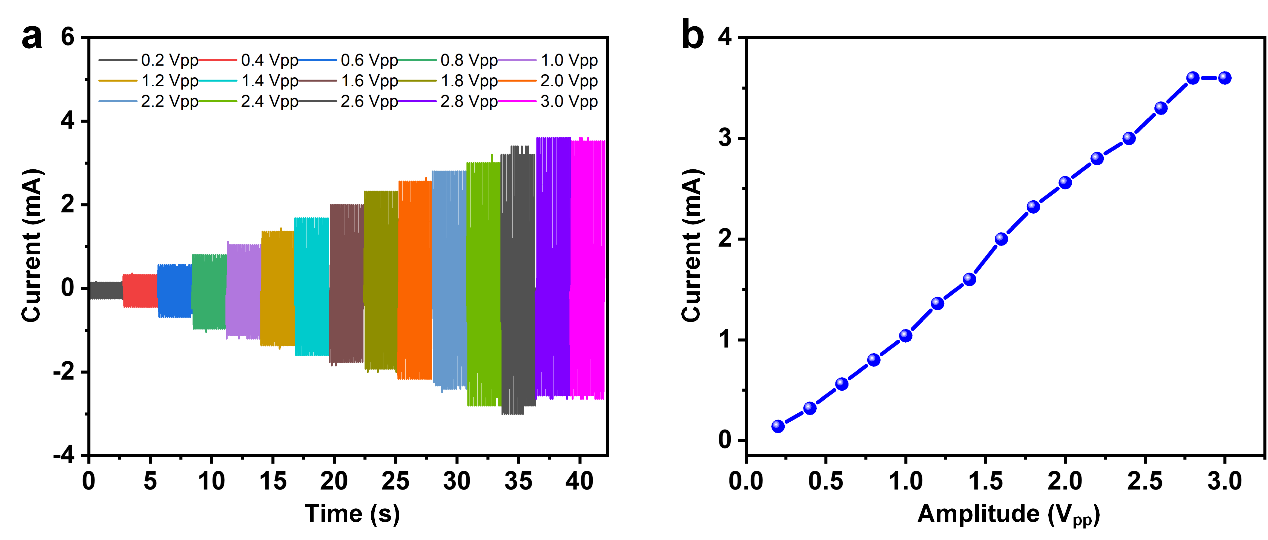


**Fig. S5** Effect of the amplitude of the external excitation from the vibration platform to the electromagnetic current output. Herein, the non-contact working state and contacted working state of the electromagnetic part of EHTE were controlled by the amplitude of the external excitation. The two contacted surfaces are silicone rubber and polyamide.

**
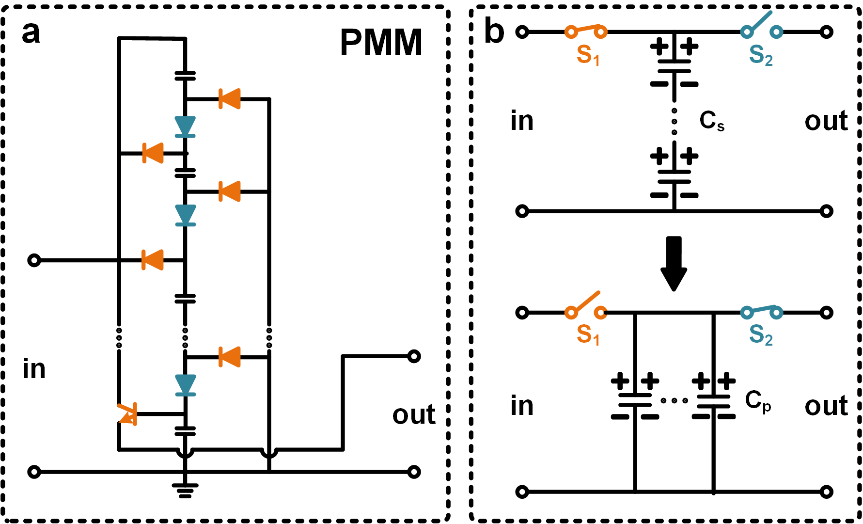
**

**Fig. S6** (a) Schematic illustration of the power management circuit (PMM). The PMM is used for the triboelectric nanogenerator (TENG) to improve huge impedance mismatch between the TENG and the energy storage device (i.e., an external capacitor). (b) The equivalent circuit model of this PMM. It is worth mentioning that this PMM is firstly developed in previous study of our group [9]. It is a transistor-controlled PMM, the transistors serve as electronic switches to automatically change the connection types of capacitors, thereby the charging state of the capacitors, i.e., series in charging state, and parallel in discharging state. When the triboelectric nanogenerator is in separating or approaching step, the blue diodes are in ON state and the orange transistors are in OFF state, the energy storage unit is charged by the triboelectric nanogenerator.


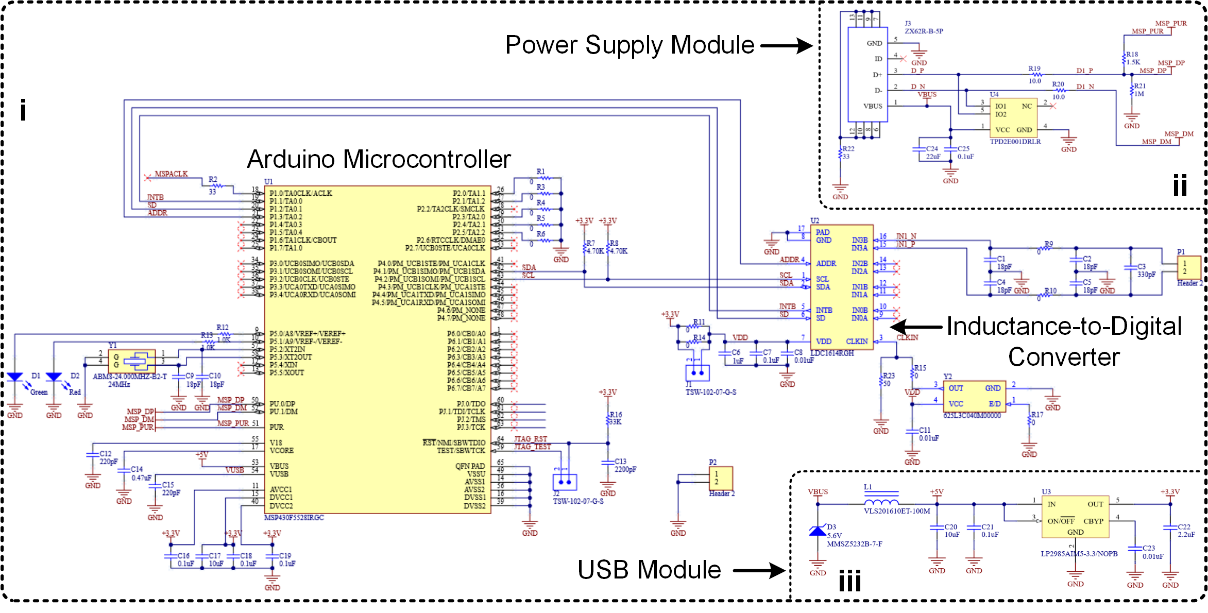


**Fig. S7** Schematic of the signal processing circuit of the inductive measurement system. It mainly includes (i) an inductance-to-digital converter and an Arduino microcontroller, (ii) the power supply module, and (iii) the USB module.


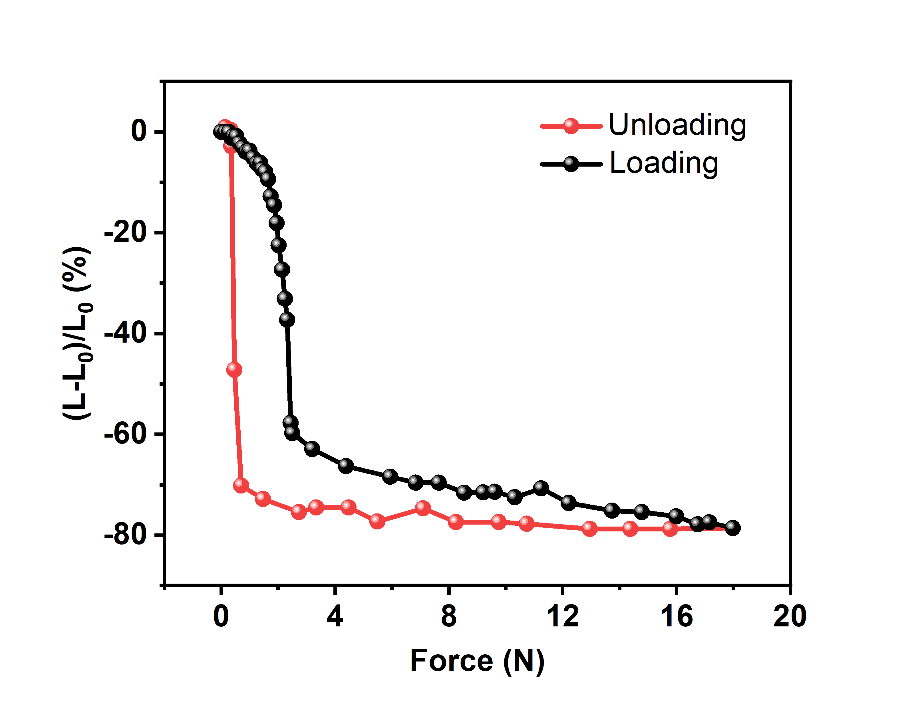


**Fig. S8** The hysteresis of the inductive pressure sensing. According to the definition of degree of hysteresis (*DH*), i.e., *DH* = (*A*_Loading_ –*A*_Unloading_)/*A*_Loading_ × 100%, where *A*_Loading_ and *A*_Unloading_ refer to the areas of the loading and unloading curves, respectively, the *DH* value of the device was calculated as 18.2%.

**
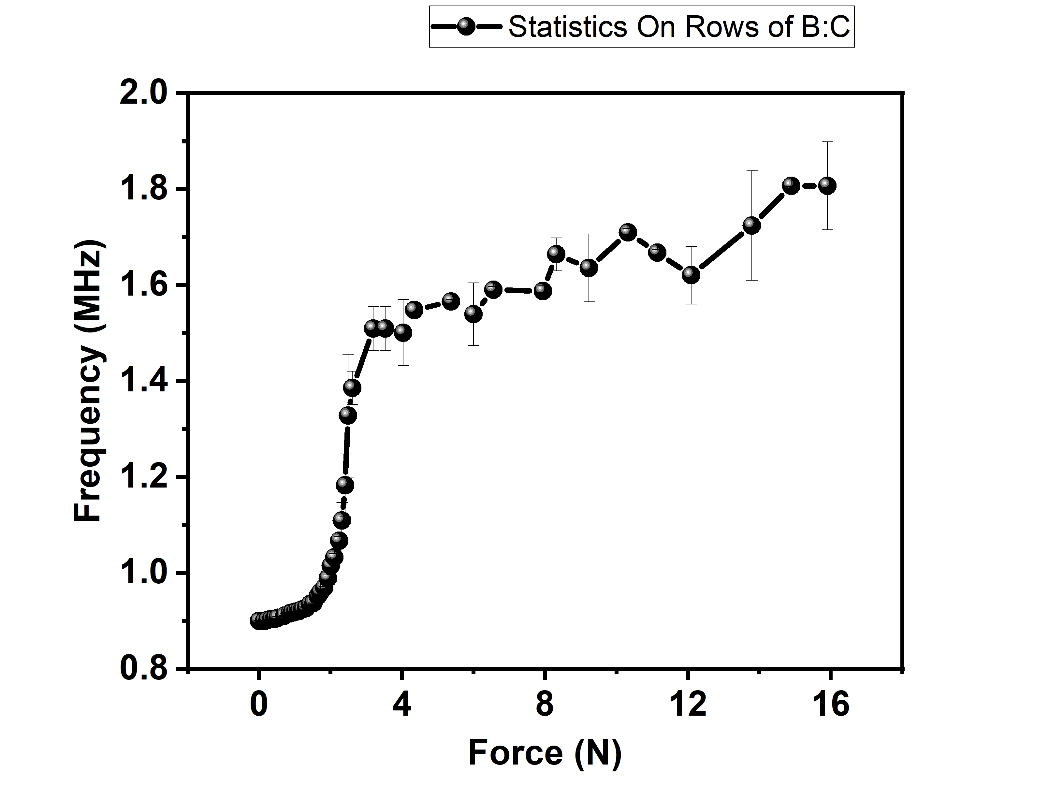
**

**Fig. S9** The oscillating frequency in LC network versus the external force. When the external pressure makes the sensing target close to the sensing coil, the coupling effect between the sensing target and the sensing coil increases, and the effective inductance of the sensing coil decreases, so that the measured frequency in the LC network gradually increases from 0.89 MHz to 1.81 MHz.


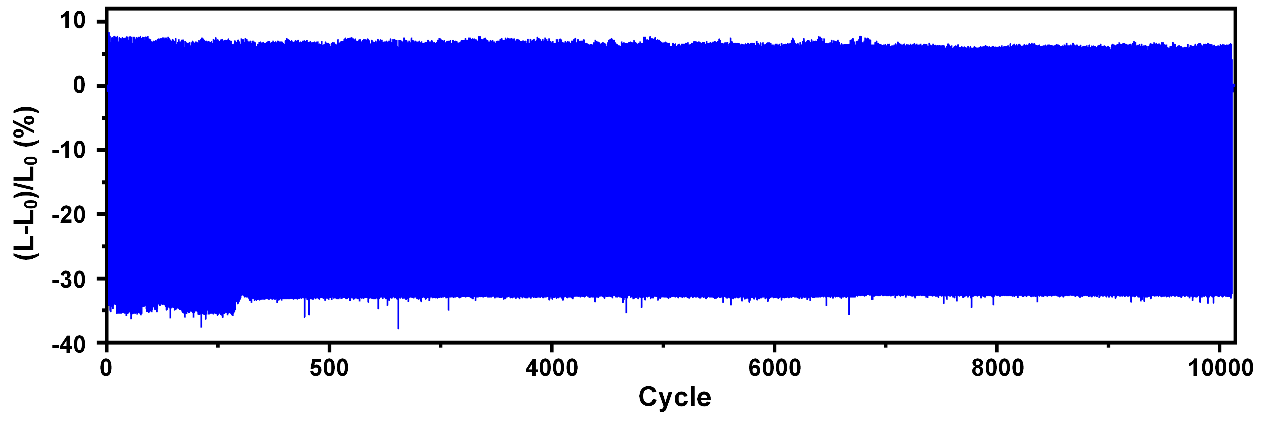


**Fig. S10** Inductive response behaviors of EHTE serving as an inductive sensor under 10400-cycle of working process, which indicates the remarkable stability of the sensing performance of the inductive sensor.


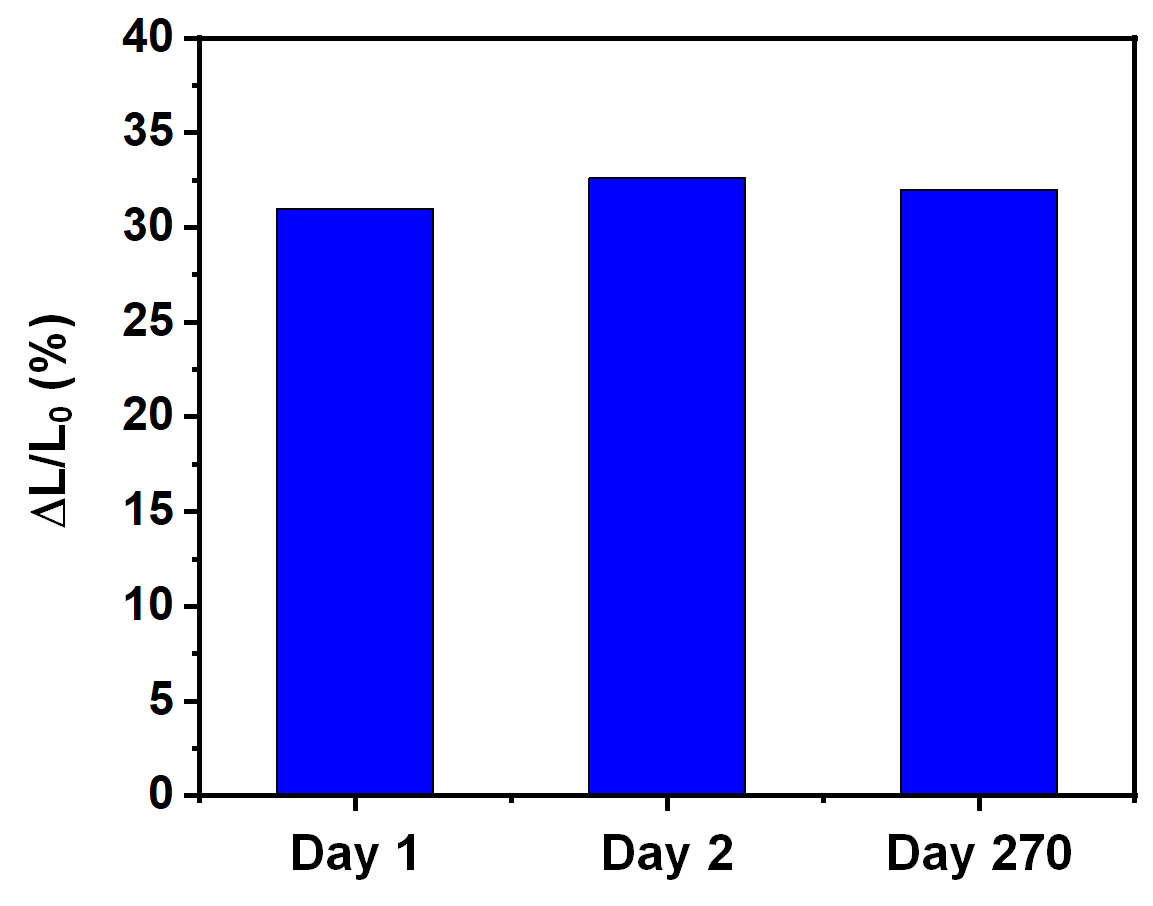


**Fig. S11** The relative inductance change of the developed EHTE in Day 1, Day 2 and Day 270. The relative inductance changes of the device only fluctuated by 5.2% and 3.2% after standing for 1 day and 9 months, respectively, indicating that the short-term and long-term stability of EHTE is remarkable.

**
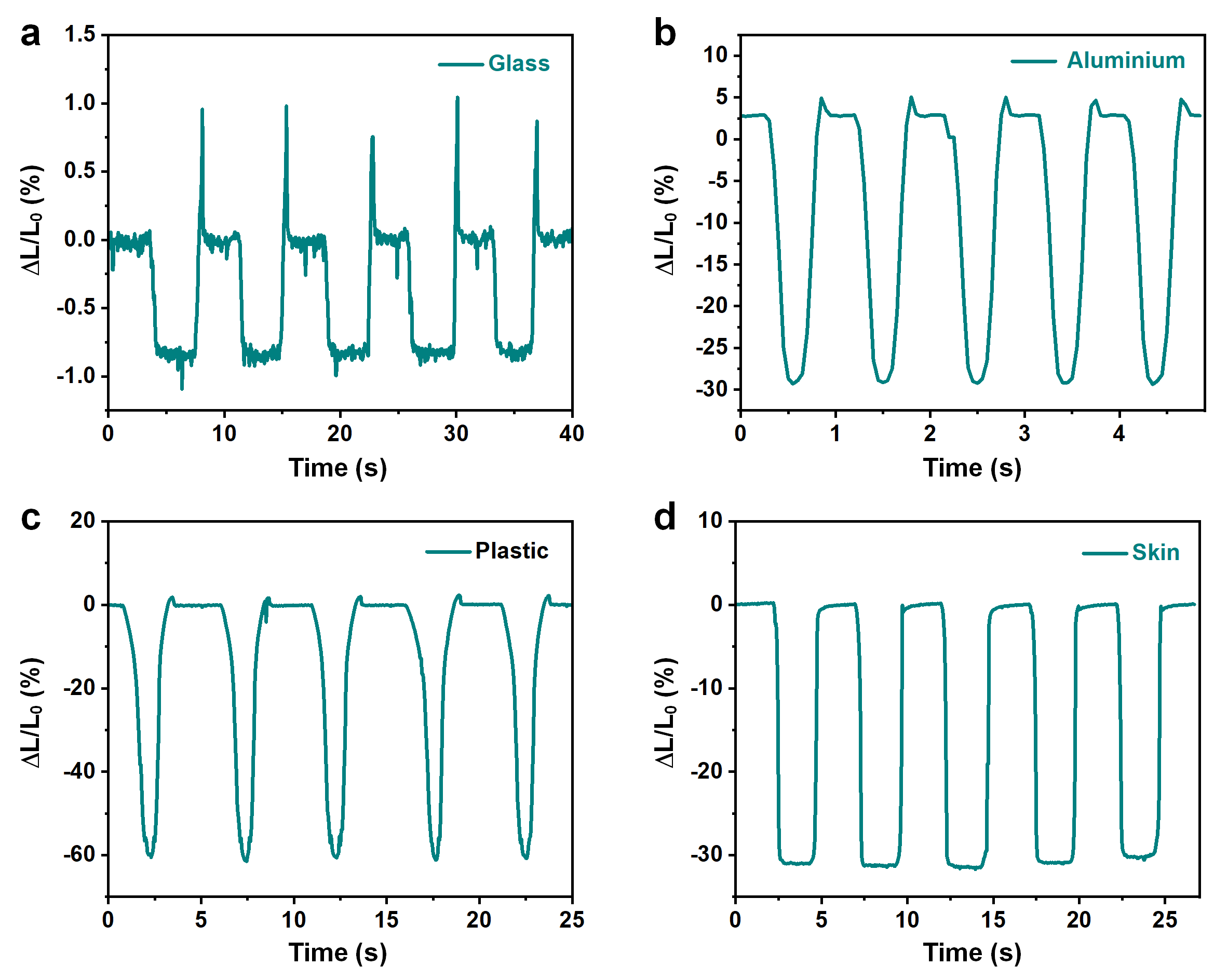
**

**Fig. S12**. The inductive pressure sensing performance of the developed EHTE to the external pressure applied by different materials, including (a) glass (b) aluminum, (c) plastic and (d) skin, with an average relative inductance change of 0.78%, 29.2, 30.1%, 60.6%, respectively.
